# Highly viable gastrointestinal *Chlamydia trachomatis* in women abstaining from receptive anal intercourse

**DOI:** 10.1038/s41598-025-85297-4

**Published:** 2025-01-10

**Authors:** Philip A. Karlsson, Mimmi Wänn, Helen Wang, Lars Falk, Björn Herrmann

**Affiliations:** 1 Section of Clinical Microbiology, Department of Medical Sciences, Uppsala University, Uppsala, Sweden; 2 Department of Medical Biochemistry and Microbiology, Infections and Immunity, Uppsala University, Uppsala, Sweden; 3 Department of Dermatology and Venereology, Linköping University Hospital, Linköping, Sweden

**Keywords:** *Chlamydia trachomatis*, Viability-PCR, Digital droplet-PCR, Gastrointestinal infection, Clinical microbiology, Infectious-disease diagnostics, Infection, Bacterial infection

## Abstract

*Chlamydia trachomatis* (CT) may employ persistence to evade antimicrobial clearance, possibly residing in the gastrointestinal tract. This study assessed the reliability of droplet digital PCR (ddPCR) in CT detection, its functionality in viability assessment, and predictions on CT transmission dynamics by combining viability PCR (vPCR) and clinical data from 52 infected women. The ddPCR showed 94% positive and 100% negative agreement with Abbott Alinity STI-M for endocervical samples, and 92% positive and 87% negative agreement in rectal samples. Viability was higher in endocervical samples (89.1%) than in rectal samples (69.4%). Samples from participants not engaging in anal intercourse, and with non-concordant multi-locus sequence typing between rectum and endocervix, had on average the highest viability in rectum, indicating a persistent population residing in the gastrointestinal tract. This study demonstrates the effectiveness of ddPCR in detecting CT, especially in samples with high inhibition or low bacterial load, suggesting its superiority over quantitative real-time PCR. These findings support that rectal CT infection can occur independently of anal intercourse, possibly through vaginorectal contamination or oral routes. High rectal CT viability, independent of endocervical infection, indicates potential gastrointestinal establishment. Understanding CT dynamics in various infection sites can provide insights into the epidemiology and pathogenesis of CT.

**Supplementary Information:**

The online version contains supplementary material available at 10.1038/s41598-025-85297-4.

## Introduction

*Chlamydia*, a genus of intracellular bacterial pathogens, are known for causing a range of diseases in animals and humans, including pneumonia, blindness and sexually transmitted infections. While antimicrobial resistance is not a primary concern for treating chlamydial infections, a unique survival tactic called “persistence” has been suggested employed to evade antimicrobial clearance^1^. *Chlamydia* spp. resides in the gastrointestinal (GI) tract of many mammalian and avian hosts without causing clinical disease, persisting indefinitely due to the immune system’s inability to clear them, and can there be shed continuously in feces^2^. The prevalence of this occurrence in humans is still unknown^3^. Antibiotic treatment, such as azithromycin, is less effective against *Chlamydia trachomatis* (CT) GI infections compared to genital infections, potentially leading to persistent GI tract infections in individuals cured of genital disease. Moreover, autoinoculation from the GI tract to the genital tract may contribute to recurrent CT infections in women following antibiotic treatment^4^. Previous research suggests that genital CT infection might occur via oral transmission through various sexual activities of other anatomical sites^5,6^, leading to persistent GI tract infections, as observed in rodents^2^. This has provided supported hypotheses around rectal CT test positivity in individuals without a history of receptive anal intercourse (RAI), and highlighted the importance of considering alternative transmission routes when interpreting CT results^3^.

Current CT-diagnostics is almost entirely based on commercial nucleic acid amplifications tests. However, the detection of CT-DNA does not necessarily signify an active infection, potentially leading to an overestimation of CT prevalence or incentives for unjustified treatment. Alternative methods such as the detection of *Chlamydia* mRNA and viability PCR (vPCR) hold promise for determining viable CT presence, nevertheless they have not yet been integrated into routine clinical practice^7^.

For quantification of bacterial load, real-time polymerase chain reaction (qPCR) has been the gold standard. As an alternative a recent study showed that the CT vPCR assay could be used in an absolute quantification system, using digital PCR (dPCR), and that dPCR could benefit result interpretation and processing time^8^. dPCRs have many advantages over qPCR, such as shorter processing time, less need for replicates and standard curves, absolute quantification and increased sensitivity – but it on the other hand lacks scalability and still relies on manual serial dilutions. A recent technology using droplet digital PCR (ddPCR) reduces the need for serial dilutions and employs a partitioning system dividing each sample up into an estimated 20 000 droplets that each generate a data point. The ddPCR has previously been shown highly efficient in detecting CT^9^, but has to the best of our knowledge, not yet been assessed in any viability assay.

Exploring innovative approaches for CT screening, this cross-sectional study investigates the potential of ddPCR alongside established clinical routine Alinity Abbott, focusing on both endocervical and rectal samples. The research aims to evaluate ddPCR reliability in CT detection, while leveraging clinical data to gain further insights into the CT transmission conundrum. Additionally, this study examines the translatability of vPCR between qPCR and ddPCR, and assesses how viability varies between different infection sites. As far as we know, this is the first time that the ddPCR is used for investigating CT viability, and how it relates to CT persistence and transmission.

## Results

In this study, patients had three locals sampled: (1) endocervix (Virocult^®^, *n* = 51), (2) the perianal area (Alinity, *n* = 40) and inner rectum (Virocult^®^, *n* = 52). Endocervix and rectum were additionally analyzed by Alinity *m* STI assay, Abbott.

### ddPCR for detection of CT

Primers and probe were designed for CT *ompA* and optimized for both ddPCR (Supplementary Fig. 1a-b) and qPCR (Supplementary Fig. 2a-c). A subset of 30 samples (57.7% of sample size) were analysed using both methods and compared to Alinity Abbott, as well as to a PCR gel electrophoresis, testing for six MLST genes (Supplementary Fig. 3**ab**). For endocervix, the ddPCR had a positive agreement with Alinity Abbott at 94% and a negative agreement at 100% (Table 1). The 6% disagreement derived from 3 samples that could not be detected in the ddPCR. One of these samples (NL55) had a Alinity Abbott ct-value of 36.2 and was not detected in any of our three methods. The remaining two (NL6 & NL15) were detected in our PCR gel electrophoresis, but not in qPCR or ddPCR (Supplementary Table 1).

**Table 1:** Endocervix agreement between Alinity Abbott and in-house ddPCR.

Endocervix	ddPCR: POSITIVE	ddPCR: NEGATIVE	Positive agreement (95% CI)	Negative agreement (95% CI)
Alinity Abbott: POSITIVE	45 (90%)	3 (6%)	94% (83–98%)	100% (18–100%)
Alinity Abbott: NEGATIVE	0 (0%)	2 (4%)		

For rectum samples, the ddPCR had a positive agreement with Alinity Abbott at 92%, and a negative agreement at 87% (Table 2). The 8% positive disagreement derived from 3 samples that could not be detected in the ddPCR. One of these (NL81) was detected in our PCR gel electrophoresis but not in the qPCR. The remaining two (NL152 & NL154) were never tested in our qPCR but did both have Alinity Abbott ct-values above 31 and did not show up in our gel (Supplementary Table 1). The 13% negative disagreement derived from two samples that were detected by the ddPCR but not by Alinity Abbott. One of these (NL56) was additionally identified in our gel, and the second one (LL131) was found weakly positive (ct 38.3) in Alinity Abbott for the perianal sample (Supplementary Table 1).

**Table 2:** Rectum agreement between Alinity Abbott and in-house ddPCR.

Rectum	ddPCR: POSITIVE	ddPCR: NEGATIVE	Positive agreement (95% CI)	Negative agreement (95% CI)
Alinity Abbott: POSITIVE	34 (65.4%)	3 (5.8%)	92% (79–97%)	87% (62–98%)
Alinity Abbott: NEGATIVE	2 (3.9%)	13 (25.0%)		

**Fig. 1:**
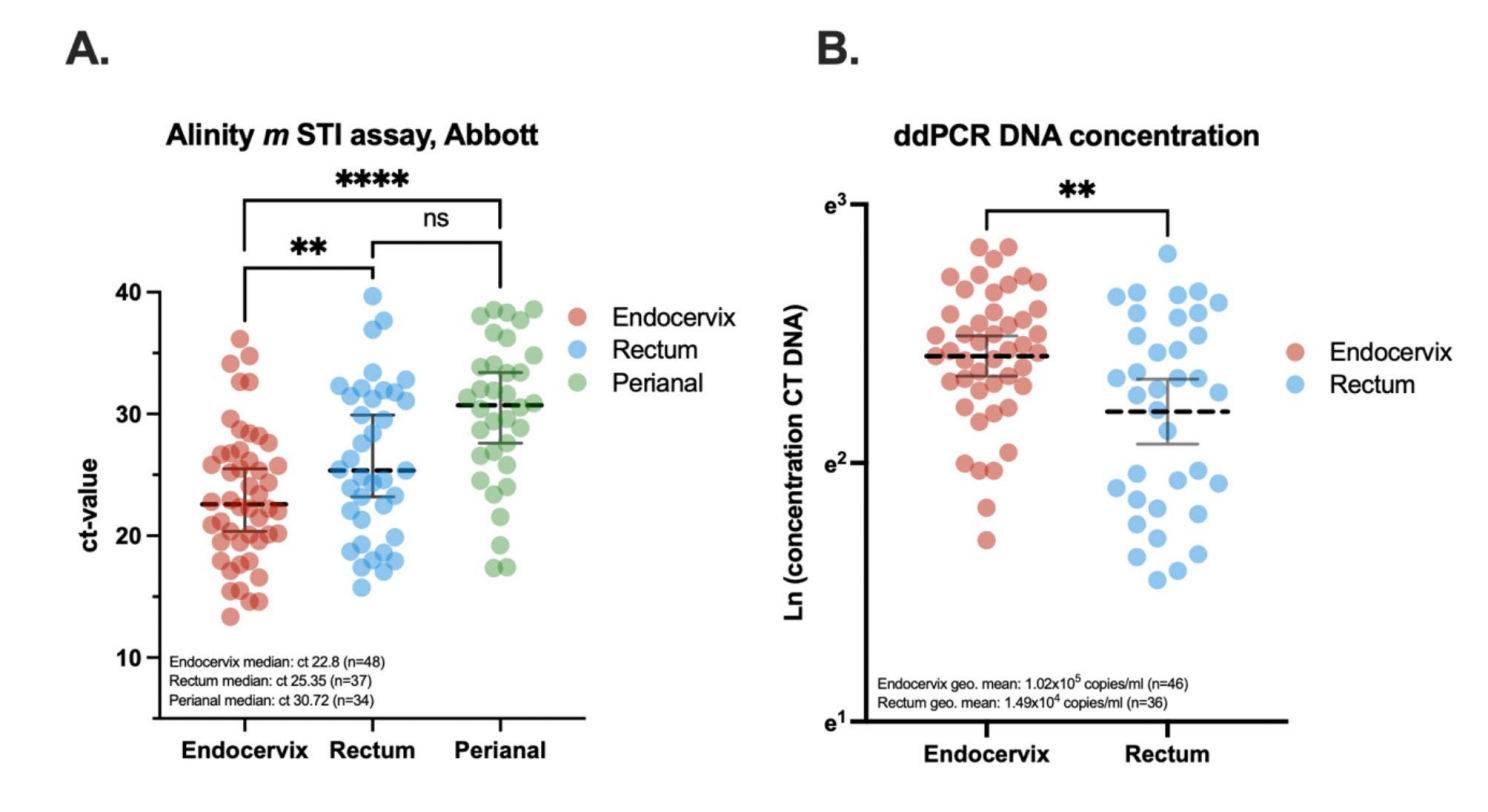
Comparison of CT-detection in endocervix (red), rectum (blue) and perianal area (green). (**A**) ct-values as acquired by Alinity *m* STI assay, Abbott. Zeros excluded for pairwise comparison and differences measured through parametric two-tailed t-test. Error bars indicate sample median with 95% confidence intervals. (**B**) Natural log (ln) of DNA concentrations acquired by in-house droplet digital PCR. Zeros excluded for pairwise comparison and differences measured through parametric two-tailed t-test. Error bars represent the geometric mean with 95% confidence intervals.

Samples derived from endocervix showed the lowest Alinity Abbott ct-values, which agreed with a higher concentration DNA detected by the ddPCR (Fig. 1a**b**). The ct-values in endocervix (x̄: 23.3, SD: ±5.6, *n* = 48) were statistically lower than both rectum (x̄: 26.2, SD: ±6.4, *n* = 37, **) and the perianal sample (x̄: 30.1, SD: ±6.0, *n* = 34, ****), but this difference was not statistically significant between perianal and rectal samples (Fig. 1a). The DNA concentration detected in endocervix ranged from 2.4 × 10^2^-2.5 × 10^7^ copies/ml (geometric mean: 1.0 × 10^5^, 95% CI: 4.4 × 10^4^-2.3 × 10^5^, *n* = 46) while ranging from 1.1 × 10^2^-1.6 × 10^7^ copies/ml (geometric mean: 1.5 × 10^4^, 95% CI: 4.8 × 10^3^-4.7 × 10^4^, *n* = 36) in rectum samples. This concentration difference was statistically significant (Fig. 1**b**, **). While the detected ddPCR DNA concentration in endocervical samples followed a normal distribution (D’Agostino & Pearson test, ns), rectal samples did not (*). A low DNA concentration in the rectum for any given sample did not correspond to a low concentration in the endocervix for the same (nonparametric Spearman, ns).

### ddPCR to assess CT viability

vPCR has previously only been performed using qPCR, hence the translatability for viability in the ddPCR system was assessed using both systems. Control CT were split in two parts, and one part heat-inactivated. Living and dead cultures were mixed in different proportions before PMA treatment and extraction. Observed qPCR ct-increases corresponded to the proportion of living bacteria in the population, and the copies/ml were similar to those previously reported (Supplementary Fig. 2**de**). Deriving viability from copy number translated into the expected ratios (Supplementary Fig. 2**f**). ddPCR measures absolute concentration, and PMA successfully inhibited free DNA from dead CT (Supplementary Fig. 4**a**) in the expected ratios (Supplementary Fig. 1**c**), resulting in good estimates of viability (Supplementary Fig. 1**d**). Using both methods, we could not identify any statistically significant difference in assessing viability using qPCR and ddPCR (Supplementary Fig. 4**b**). This was also performed for a subset of clinical strains with similar agreement (data not shown).

### Viability of CT from endocervix and rectum

**Fig. 2:**
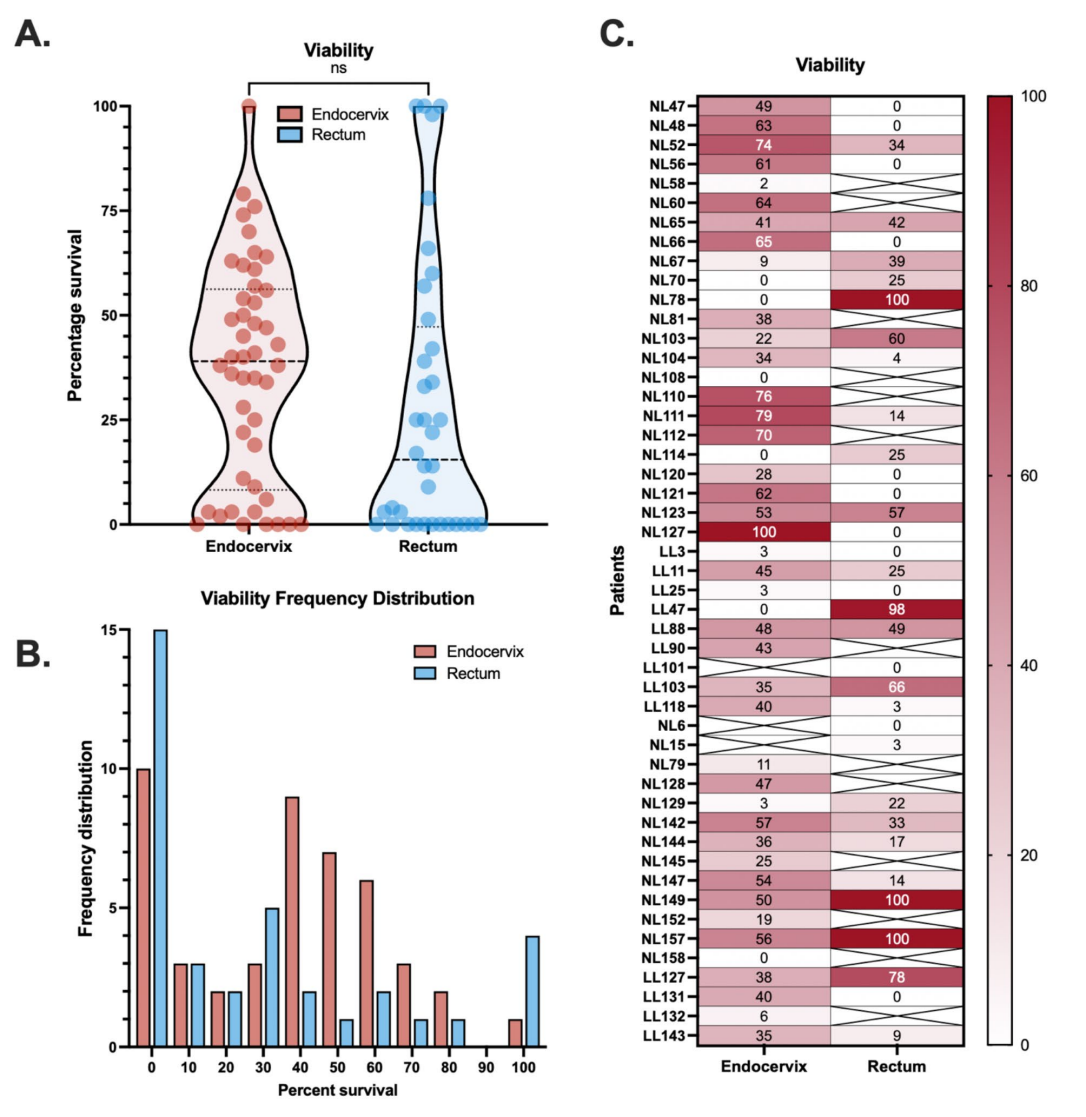
Viability of CT in endocervix and rectum as measured by ddPCR. Samples were divided in two, and one part treated with photoreactive propidium monoazide (PMA). The concentration CT in PMA-treated samples were divided by the uninhibited sample and presented as a percentage of viability. (**A**) Viability of CT from endocervix (red) and rectum (blue), plotted in a violin graph showing the median (dotted line, thick), and the 25%/75% percentile (dotted line, thin). Significance was measured with a paired two-tailed parametric t-test. (**B**) Cumulative frequency of measured viability with automatic binning (10% per group). Endocervix (red) and rectum (blue), and the y-axis indicates the percentage of the corresponding sample being found at what viability (x-axis). (**C**) Heat-map showing the viability across all analyzed samples. Color grading goes from 100% viability (dark red) to 0% viability (white), and the exact measured viability is found within each box. Missing values (negative ddPCR for PMA untreated sample) are represented by a cross in the box.

Samples received and tested were overall viable (Fig. 2). In total, we could only identify five (10.9%) non-viable endocervical samples, while eleven (30.6%) non-viable rectum samples were found. Endocervix and rectum additionally contained four (8.7%) and three (8.3%) samples, respectively, with a viability below 5%. Rectum samples were overrepresented among both 0% and 100% viable samples (Fig. 2**ab**). Given the sample being viable, endocervix had a median per-sample viability of 39% (x̄: 36.6%, SD: ±26.3%, *n* = 46) and rectum a median per-sample viability of 15.5% (x̄: 28.3%, SD: ±33.5%, *n* = 36). The per-sample viability in rectal samples were lower, but the deviations between the means were not statistically significantly different (Fig. 2**a**). Viability of endocervical samples followed a normal distribution, while rectal samples did not (Fig. 2**b**, *). At further inspection, the rectal samples included a higher proportion of extreme values (e.g., 0% and 100%). Viability between endocervix and rectum did not correlate, but rather we found a trend in the opposite direction. High viability in the rectum appeared to be associated with low viability in endocervix, signifying sporadic contamination (nonparametric Spearman, *r* = -0.24, ns, Fig. 2**c**). The lack of statistical significance indicates that this pattern is not consistent; occasionally, high viability is observed in both sites and instead proposing active infections in both anatomical locations.

### Assessment of viability in relation to clinical data

ddPCR results were assessed in relation to collected clinical data. In total 72.5% (*n* = 51) of rectal samples were positive. Among the 51 patients, 33.3% answered that they had participated in RAI, while 66.6% answered that they had not. The proportion of positive rectal samples were higher in patients answering that they had participated in RAI (88.2% vs. 64.7%, Table 3). Rectum CT viability across the groups were consistently higher (t-test, ****) in the group participating in RAI (x̄: 31%, SD: 0.35, *n* = 15) compared to those not participating (x̄: 26%, SD: 0.33, *n* = 26). However, there were a few exceptions. For instance, the viability of identified rectal CT was 0% in one patient who had participated in RAI but had reported an anovaginal wiping direction after defecation (NL6). This patient did not have any detectable CT in endocervix using ddPCR (routine ct-value endocervix: 28.4). Only one patient (NL104) who had been reporting RAI showed a lack of CT MLST concordance between endocervix and rectum, and here the viability was very low (4%, *n* = 1). This is in stark contrast to patients reporting no RAI, where 35.3% (*n* = 17) showed a lack of MLST concordance between rectum and endocervix. In fact, the viability among non-concordant CT found in rectum had the highest viability (41.7%, *n* = 6) of all rectal samples. All samples contributing to the 41.7% viability was from patients reporting receptive oral ejaculation. The highest CT concentration was consistently found in samples showing concordance in MLST types between endocervix and rectum, while the lowest concentrations were found among patients showing lack of concordance, or an anovaginal wiping pattern. While CT concentration differed within this subgroup, it was not significantly different overall when comparing RAI with no RAI (t-test, ns).

**Table 3:** ddPCR assessment of rectal CT with regards to clinical data.

	Answered “receptive anal intercourse within the last year”			Answered “no receptive anal intercourse within the last year”		
	Number	Conc (c/ml)	Viability (%)	Number	Conc (c/ml)	Viability (%)
Positive rectum	15	3.7 × 10^5^	31.3	22	9.5 × 10^5^	26.1
Negative rectum	2	0	NA	12	0	NA
Receptive oral ejaculation	15	3.6 × 10^5^	31.4	19	9.3 × 10^5^	28.3
No receptive oral ejaculation	2	2.0 × 10^4^	30.0	11	2.8 × 10^5^	25.9
Vaginorectal wiping direction after defecation (front-to-back)	13	4.2 × 10^5^	36.1	26	7.6 × 10^5^	33.3
Anovaginal wiping direction after defecation (back-to-front)	1	1.8 × 10^4^	0	5	2.4 × 10^4^	5.0
Concordance MLST	10	3.7 × 10^5^	33.4	11	1.5 × 10^6^	15.0
No concordance MLST	1	1.6 × 10^4^	4.0	6	4.8 × 10^3^	41.7

## Discussion

This study has evaluated the reliability of ddPCR for CT detection, comparing vPCR methods, and examining CT viability across infection sites. The most important finding is that ddPCR is a sensitive and reliable method for quantitative CT detection. The use of vPCR supports that:

1. Non-viable CT DNA from endocervix may reach rectum as contamination.
2. Endocervical CT may colonize rectum and establish infection.
3. GI CT-infection may be present independent from endocervical infection.

The application of ddPCR in screening for CT yielded promising results, with a high positive agreement and a slightly lower negative agreement between ddPCR and Alinity Abbott. Discrepancies derived partly from certain samples being undetected in ddPCR, but mainly from ddPCR-positive rectal samples shown as weakly positive, or negative, in Alinity Abbott. ddPCR sensitivity has previously been reported to outperform qPCR^10,11^, and the heightened sensitivity might be required in samples with high degree of inhibition^8,12,13^, especially when the bacterial load is low, or in cases of rectal intermittent CT shedding^4,14^. ddPCR can encounter detection losses in samples with too high concentration of DNA, where droplet saturation would prevent separation of positive and negative droplets^15,16^. Sample dilution was in our study required to increase sensitivity of the method, but sample digest^17^, or dual-target screening^18,19^, could have been additional approaches to increase assay sensitivity. Viability assessment in the ddPCR system was compared to qPCR, demonstrating similar results and confirming the translatability of viability between the two methods, in agreement with the translatability of dPCR^8^.

Endocervical samples exhibited statistically significantly lower ct-values compared to rectal and perianal samples, consistent with higher DNA concentrations detected by ddPCR, validating the utility of ddPCR. Established genital CT would arguably increase the likelihood for vaginorectal contamination^20^. The variation in ct-values within sampling sites remains larger than the differences between them, and as previously suggested, viability might be a better indicator for relevance to infection^21,22^.

Most participants with rectal CT had not participated in RAI, adding to the increasing evidence that RAI alone is not a sufficient indicator for rectal CT^23,24^. As previously introduced, there are reasons to suspect genital, or even oral, CT infection as a risk for chronic GI-tract colonization^2–4^. GI-colonization might in turn lead to chronic or recurrent genital infection and pathogenicity^5,25^, something which has been suggested related to the dynamic regulation of plasmids^26^. While our data at large augments this hypothesis, our findings provide little clarity in how CT reaches the GI-tract. While participants partaking in receptive oral ejaculation had a slightly higher rectal CT concentration, this was not statistically significant. While vaginorectal contamination might appear like the most straightforward route, a recent study demonstrated that GI CT in men is no different from women having sex with men^27^. This might support the importance of an oral route, or anilingus activity, as GI CT is even found among MSM not partaking in RAI^6,28–31^. For cases with different MLST types, rectal positivity could speculatively come from past exposure that settled in the GI tract, having multiple sex partners, or one partner carrying different MLST types.

Overall, CT in the samples tested were highly viable. While our samples have a generally higher viability than previously reported^32^, our data similarly strengthens the argument that CT in rectum are largely viable, and not just a contamination of nucleic acid. Being viable in endocervix did however not correlate with being viable in rectum, and this contrasts with previous findings^33^. A disagreement between endocervix and rectum insinuates potential independent occurrences of CT infection between the two sites and highlights that vaginorectal DNA-contamination indeed can occur. That the rectal viability was not normally distributed, grouping at high or negligibly low viability, additionally hints at genuine rectal/gastrointestinal establishment vs. occasional cross-contamination. There were several instances of viable rectal CT with no living endocervical CT. This would imply that this anatomical site might have cleared the infection, or that anovaginal contamination is responsible for the positive endocervix result. Higher rectal CT viability was found in patients reporting RAI, aligning with expectations and corroborating our dataset.

The highest rectal CT viability was identified in a subset of samples from patients reporting no receptive anal intercourse and having non-concordance (MLST) between rectum and endocervix. Different ST-types identified in endocervix and rectum suggests the presence of a gastrointestinal CT strain distinct from the one causing the endocervical infection. This may arise when a person has multiple sex partners or a previous infection that never cleared, leading to GI establishment. Furthermore, the low CT concentration observed in non-concordant, highly viable rectal samples among patients abstaining from RAI, might propose a potential persistent low-level population^2^. To the best of our knowledge, this is the second study demonstrating non-concordance between rectum and endocervix^34^, indicating the importance of the human GI-tract in chlamydia disease.

The use of ddPCR combined with vPCR indicates that contamination with non-viable DNA occurs and results in false positive CT tests in routine diagnostics, and detection of GI strains of unknown significance may occasionally also be detected. These results lead to the ongoing debate about indications for testing and handling of CT infections, especially asymptomatic infections^35,36^. Thus, there is a need for more accurate methods in routine diagnostics. However, current ddPCR and vPCR methods are too labor-intensive and expensive to be used in clinical high-volume testing.

A strength with this study is the use of complementary and adequate methods to investigate the complex transmission dynamics of CT infections. A clear weakness of the study is the relatively few patients included in the study. Although our findings support different and parallel transmission routes, a larger data set would have increased the strength.

In summary, ddPCR shows promise in CT screening, with high agreement rates compared to Abbott Alinity. Viability assessment using ddPCR is comparable to qPCR, offering an efficient workflow and expediating result interpretation. The elevated rectal viability, low DNA concentration and increased non-concordance among a group of patients not engaging in anal intercourse suggest the presence of a distinct low-frequency gastrointestinal CT strain, underscoring the complexity of CT transmission dynamics and warrants further investigation into alternative transmission routes.

## Methods

### Sample collection

This study is a multicenter cross-sectional study investigating CT in female patients seeking care at included Swedish STD clinics (Norrköping, Linköping, Jönköping).

Ethical permit was approved 12th of May 2019 by the Swedish Ethical Review Authority (Dnr: 2019 − 01541). Informed consent to participate in the study was obtained from patients included in the study. This study was performed in line with the principles of the Declaration of Helsinki.

All methods used were performed in accordance with the relevant guidelines and regulations. Between September 2019, and August 2022, 313 patients were enrolled in the study. In total, 58 (18.5%) patients tested positive (endocervix) for CT. Due to the necessity of samples never experiencing freeze-thaw cycles before vPCR, six samples were excluded, resulting in 52 CT-positive patients. Patients fulfilling inclusion criteria, had the following swab samples collected in the following order:

1. One Alinity multi-Collect Specimen Collection Kit with pierceable cap (Abbott 09K12-004), perianal (and sphincter area), for qPCR.
2. One Alinity multi-Collect Specimen Collection Kit with pierceable cap, rectum, for routine diagnostics (collected using a pediatric sized proctoscope).
3. One Virocult (Sigma, MW951S2ML) tube, rectum, for vPCR/ddPCR/qPCR, sequencing (collected using a paediatric sized proctoscope).
4. One Virocult tube, endocervix, for vPCR/ddPCR/qPCR, sequencing.
5. One Alinity multi-Collect Specimen Collection Kit with pierceable cap, endocervix, for routine diagnostics.

Swabs (1, 3 & 4) were immediately frozen at -80 °C and eventually transported on dry ice until DNA extraction. In this study, Alinity results were used to compare and ensure the reliability of ddPCR, and all conclusions are made based on ddPCR data.

### vPCR and DNA extraction

Ice-thawed samples were pulse vortexed (10 s) and aliquoted (400 µL) into two microcentrifuge tubes. One µL of 20 mM photoreactive propidium monoazide (PMAxx™, Biotium) was added to one of the aliquots (PMA+) and vortexed for 15 sec^37^. Both PMA + and PMA- samples were incubated for 10 min at 4 °C for downstream viability PCR (vPCR), as previously explained for CT^7^. PMA + samples were illuminated with the PMA-Lite™ LED Photolysis Device (Biotium) for 10 min, followed by immediate storage at -20 °C until extraction. A total of 300 µL PMA+/- sample was used for DNA extraction and eluted in 300 µL elution buffer (QIAamp^®^ DNA Mini Kit, Qiagen).

### PCR gel electrophoresis and MLST typing

PCR was performed as previously described^38^, targeting six genes: *hctB*, *CT058*, *CT144*, *CT172*, *pbpB* (*CT682*) and *ompA*. Gene amplicons were visualized on gel and in this study used as an additional comparison measure to qPCR and ddPCR. If negative, a set of additional inner primers were tested (nested PCR). If visible, PCR products were treated with PCR clean-up, containing Exonuclease I and Shrimp Alkaline Phosphate (ExoProStar™ Amersham Bioscience, or A’SAP ArcticZymes^®^). PCR products were sequenced (Sanger-sequencing, Macrogen Europe Bv, Amsterdam, The Netherlands) and used for multi-locus sequence typing (MLST). MLST was in this study used to control for concordance between collected rectal and endocervical samples. For a full list of PCR/sequencing primers, see Supplementary Table 2.

### Assay optimization

PMA+/- samples were analyzed using the probe-based QX200 Droplet Digital™ PCR (ddPCR, Bio-Rad) system. Controls, and a subset of clinical samples, were additionally analyzed using CFX96 quantitative real-time PCR (qPCR) to validate viability. All samples were compared to conventional PCR and gel electrophoresis. Primers and probe validation was performed using synthetic CT LGV II 434 DNA (AMPLIRUN^®^, Vircell) and an in-house extraction control (150916/KV 181206BH). Technical validation of the vPCR was performed using mixed live-dead (heat-inactivated, non-infectious) CT ATCC VT-885 (Table 4). A negative control (*Haemophilus influenzae*), negative template control (NTC) and the extraction control were included throughout.

**Table 4:** Control strains.

Strain	Culture collection designation	Use in study
*Chlamydia trachomatis* Serovar D	ATCC VR-885	Viability gradient control
*Chlamydia trachomatis* LGV II 434	ATCC VR-902BD	AMPLIRUN^®^ Vircell copy number calibration
*Chlamydia trachomatis* 150,916/KV 181206BH		In-house extraction control

For all amplification in qPCR/ddPCR systems, previously validated primers and probe were used, targeting the CT major outer membrane protein (MOMP) (Table 5)^39^. Primers (Eurogentec) were validated in our systems, and the protocol was slightly optimized to work with the ddPCR system.

**Table 5:** Primers and probe.

Name	Sequence	Fluorophore/quencher
HJ-MOMP-1 (forward)	5ʹ-GACTTTGTTTTCGACCGTGTT-3ʹ	
HJ-MOMP-2 (reverse)	5ʹ-ACARAATACATCAAARCGATCCCA-3ʹ	
MGB-MOMP probe	5ʹ-ATGTTTACVAAYGCYGCTT-3ʹ	VIC/NFQ

### qPCR & ddPCR

A conjoint qPCR/ddPCR primer-probe mixture (PPM) was prepared containing 840 nM primer and 100 nM probe (MGB: minor groove binder, VIC: 2’-chloro-7’-phenyl-1,4-dichloro-6-carboxyfluorescein, NFQ: non fluorescent quencher). qPCR reactions were prepared in 25 µL reaction volumes, containing 10 µL template, 12.5 µL TATAA GrandMaster^®^ Mix and 2.5 µL PPM, and results were analyzed in triplicates. ddPCR reactions were prepared in 22 µL reaction volumes, containing 12.5 µL ddPCR™ Supermix, 2.5 µL PPM and 7 µL template, with results analyzed in singles. PCRs were run according to Table 6. When analyzing clinical samples, negative control, NTC, extraction control, copy number control (Vircell) and a subset of the viability gradient controls were included on every plate. The sample was re-run if the total droplet count was below 15,000, the average amplitude of negative droplets was above 1000 or the average separation between negative and positive droplets was below amplitude 500, the sample was re-run. If the total number of negative droplets were below 500, or the total number of positive droplets was below five, the sample was diluted (1:10, 1:100 or 1:1000) and analyzed again. Samples with values within these criteria were not controlled again.

**Table 6:** Polymerase chain reaction.

	qPCR TATAA GrandMaster^®^ Mix	ddPCR ddPCR™ Supermix	
HotStart	95 °C, 15 min	95 °C, 15 min	
Denaturation	95 °C, 15 s	95 °C, 15 s	42x cycles
Annealing/elongation	60 °C, 1 min	56.5 °C, 1 min with 2 °C/s ramp-up	
Hold	4 °C, indefinitely	4 °C, indefinitely	

### Analysis

Positive and negative agreement are neutral assessments of two methods and are used in place of positive and negative predictive values to compare Alinity Abbott with in-house established ddPCR. While Alinity Abbott defines positive results at cycle number (CN), cycle threshold (ct) is used throughout this paper for consistency. qPCR and ddPCR quantify relative and absolute values, respectively, hence results were compared following conversion into binary data and analyzed in a contingency table. Lognormal distribution was analyzed using D’Agostino & Pearson test and correlation was analysed using two-tailed nonparametric Spearman (r for every pair, row exclusion at missing values). Alinity Abbott ct-values were compared between sampling sites using a paired two-tailed parametric t-test. DNA concentrations ranged from 10^1^ to 10^8^ copies/ml (not normally distributed), distorting arithmetic means. Hence, ddPCR values were transformed (natural log) and compared (parametric t-test) based on geometric means. Zeros were excluded in both pairwise comparisons. For ddPCR, viability was calculated using the simple ratio between PMA- and PMA + quantifications. qPCR performs with relative quantification and copy number (log10 copies/ml) was derived using a calibration curve. To assess viability, the difference in copy number was set in proportion to an uninhibited sample (**Supplementary data**). qPCR values were reported as one average from three replicates, while ddPCR values were reported as singles. Data analysis and presentation of ddPCR droplet data were performed using QuantaSoft Software v1.7, while statistical analysis and remaining data presentation were performed using GraphPad Prism v10.2.1. A statistically significant difference was identified at p-values smaller than 0.05 with * denoting < 0.05, **<0.01, ***<0.001, and ****<0.0001. No statistical significance was denoted with ns (not significant).

### Formula viability

$$\:{\%\:viability}_{ddPCR}=\frac{{PMA}^{+}\left[DNA\right]\:}{{PMA}^{-}\left[DNA\right]}\times\:100$$

$$\:{\%\:viability}_{qPCR}=\frac{100\:}{{10}^{({PMA}^{-}log10\:copies/ml)-{(PMA}^{+}log10\:copies/ml)}}$$

## Electronic supplementary material

Below is the link to the electronic supplementary material.

Supplementary Material 1

## Data Availability

Data is provided within the manuscript or supplementary information files.
