# Supplementary material for: Highly viable gastrointestinal Chlamydia trachomatis in women abstaining from receptive anal intercourse
Source: Sci Rep. 2025 Jan 10;15:1641. doi: 10.1038/s41598-025-85297-4 (PMC11724036; doi:10.1038/s41598-025-85297-4)
Supplement: Supplementary file 1 — Supplementary Material 1 [file 41598_2025_85297_MOESM1_ESM.docx]

**Highly viable gastrointestinal Chlamydia trachomatis in women abstaining from receptive anal intercourse
Philip A. Karlsson, Mimmi Wänn, Helen Wang, Lars Falk, Björn Herrmann**

**Supplementary information**


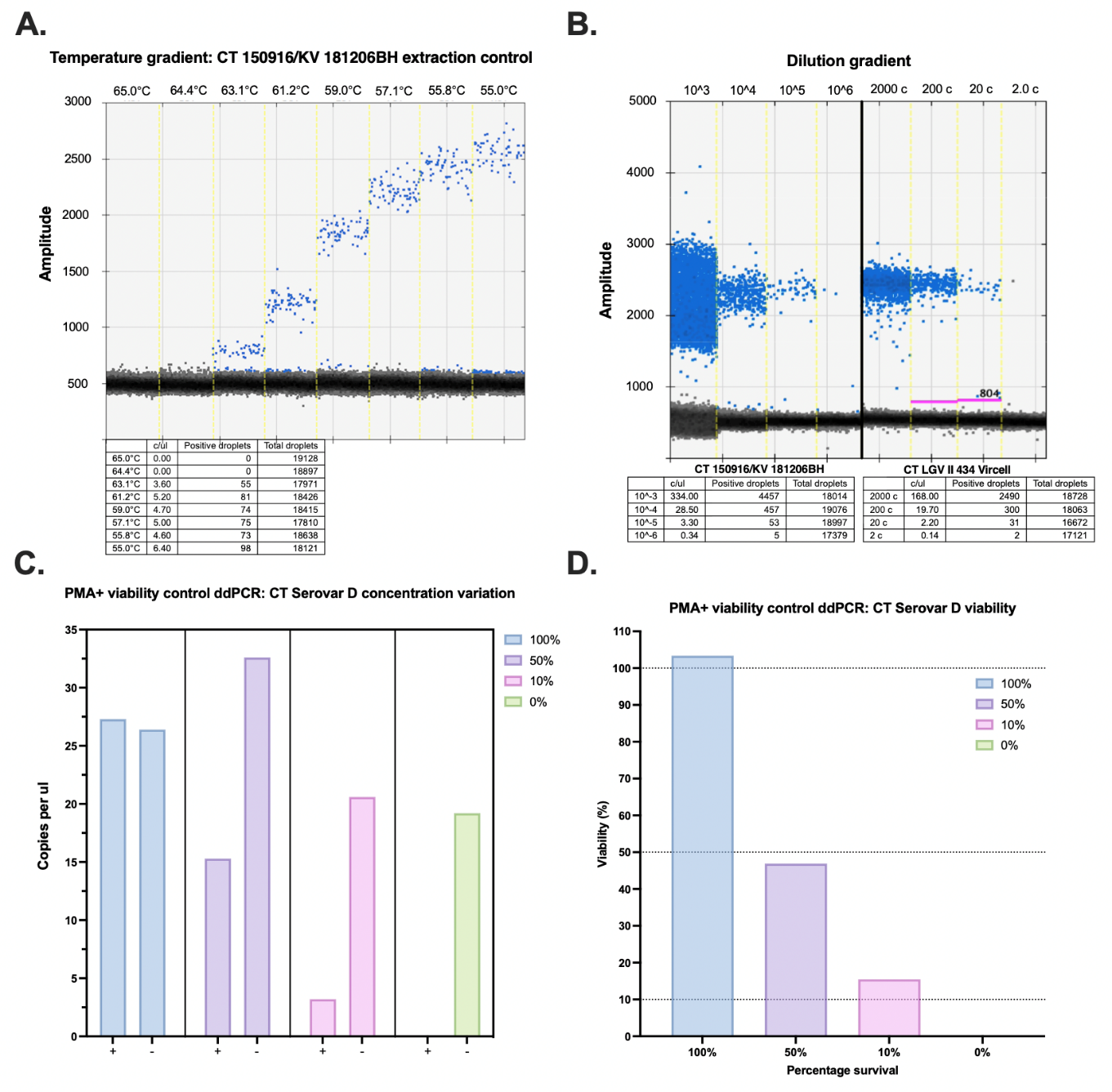


**Supplementary Figure 1.** **Optimization of *ompA* primers and probe for use in screening and viability PCR using droplet digital PCR.** **A.** Annealing temperature gradient ranging from 65.0°C to 55.0°C. Table indicates temperature, copies (c) per μL, positive and total number of droplets. **B.** Ten-fold dilution gradient at 56.5°C for a CT extraction control (CT 150916/KV 181206BH, left) and a Vircell copy number control (CT LGV II 434, right). Pink lines represent manual thresholds where automatic software detection failed to include positive droplets. **C.** Copies per μL in samples (viability control CT Serovar D) treated with photoreactive propidium monoazide (PMA) (+) or without treatment (-). Color indicates the percentage of living CT in the sample (dead proportion killed by heat inactivation), with 100% (blue), 50% (purple), 10% (pink) and 0% (green) survival. **D.** Viability graph showing estimated viability from dividing the proportion of detected dead cells (PMA+) against the total population (PMA-, uninhibited). Color indicates survival, with 100% (blue), 50% (purple), 10% (pink) and 0% (green).


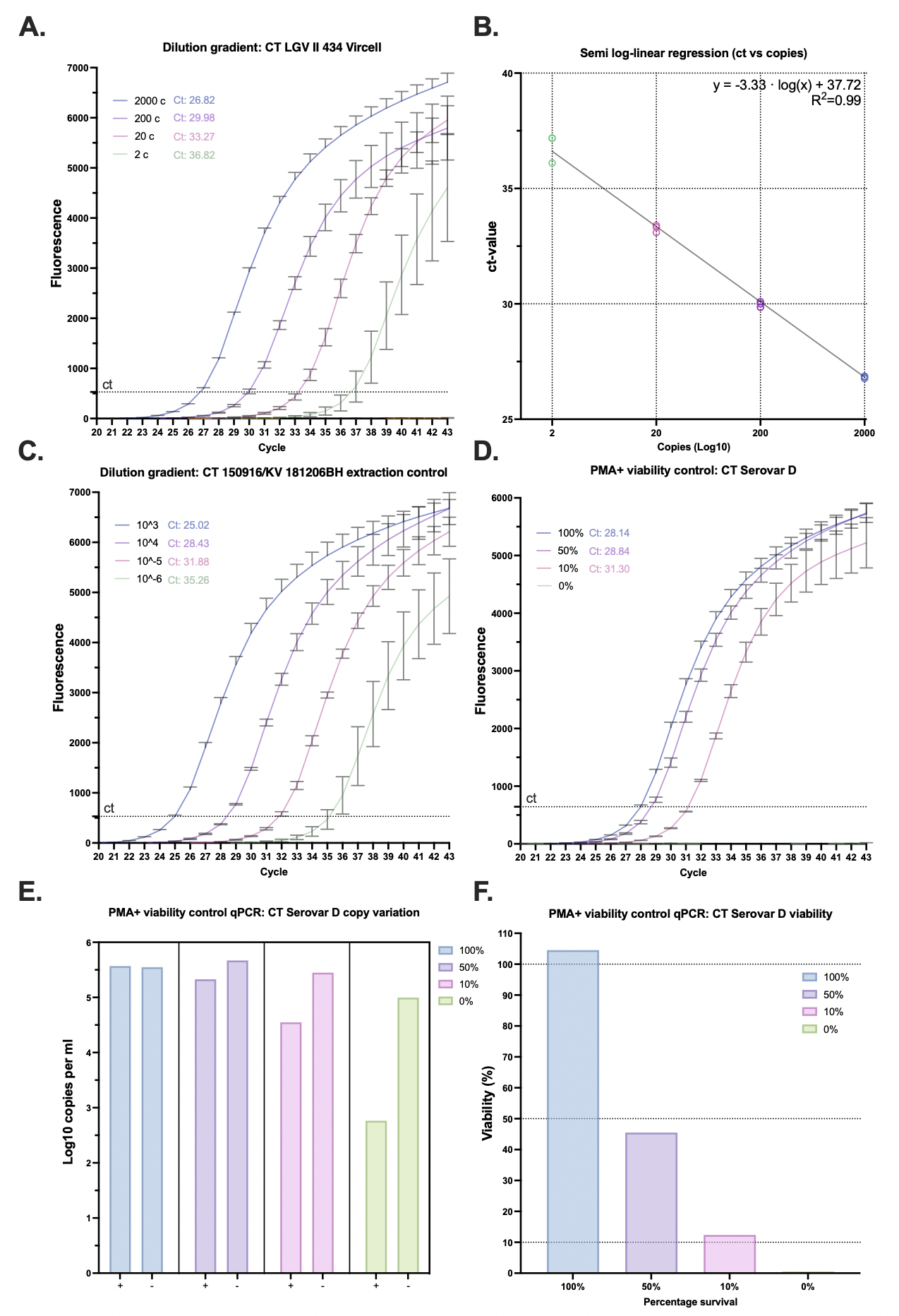


**Supplementary Figure 2. Optimization of *ompA* primers and probe using quantitative real-time PCR for comparative use in screening and viability PCR using droplet digital PCR.** **A.** Ten-fold dilution gradient at 60°C (as described by Jalal et al.) for a Vircell copy number control (CT LGV II 434). **B.** Graph showing amplification efficiency at each 10-fold dilution. **C.** Ten-fold dilution gradient at 60°C for a CT extraction control (CT 150916/KV 181206BH), indicating similar efficiency as for the copy number control. **D.** Amplification graph of samples (viability control CT Serovar D) treated with photoreactive propidium monoazide (PMA). Color indicates the percentage of living CT in the sample (dead proportion killed by heat inactivation), with 100% (blue), 50% (purple), 10% (pink) and 0% (green) survival. **E.** Log10 copies per ml in samples (viability control CT Serovar D) treated PMA (+) or without treatment (-). Color indicates the percentage of living CT in the sample, with 100% (blue), 50% (purple), 10% (pink) and 0% (green) survival. **F.** Viability graph showing estimated viability derived from the difference in the proportion of detected dead cells (PMA+) and total cells (PMA-, uninhibited). Color indicates survival, with 100% (blue), 50% (purple), 10% (pink) and 0% (green).


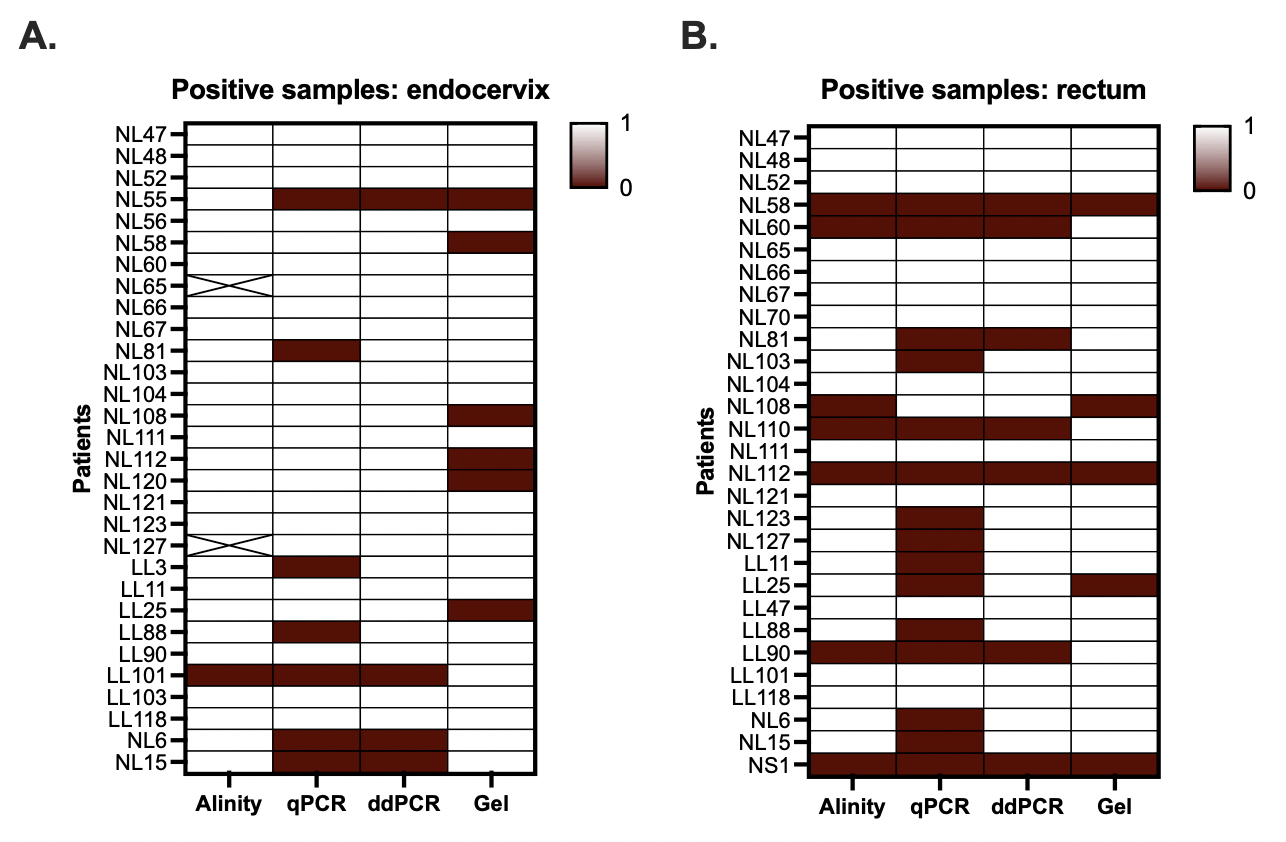


**Supplementary Figure 3. Binary representation of positive outcome for *C. trachomatis* using different methods.** Color indicates positive (white) and negative (red) results in either Alinity Abbott (Alinity), in-house qPCR (qPCR), in-house ddPCR (ddPCR) and in-house conventional PCR with gel electrophoresis (gel). Crossed out boxes indicate positive results but where Ct-values are missing.


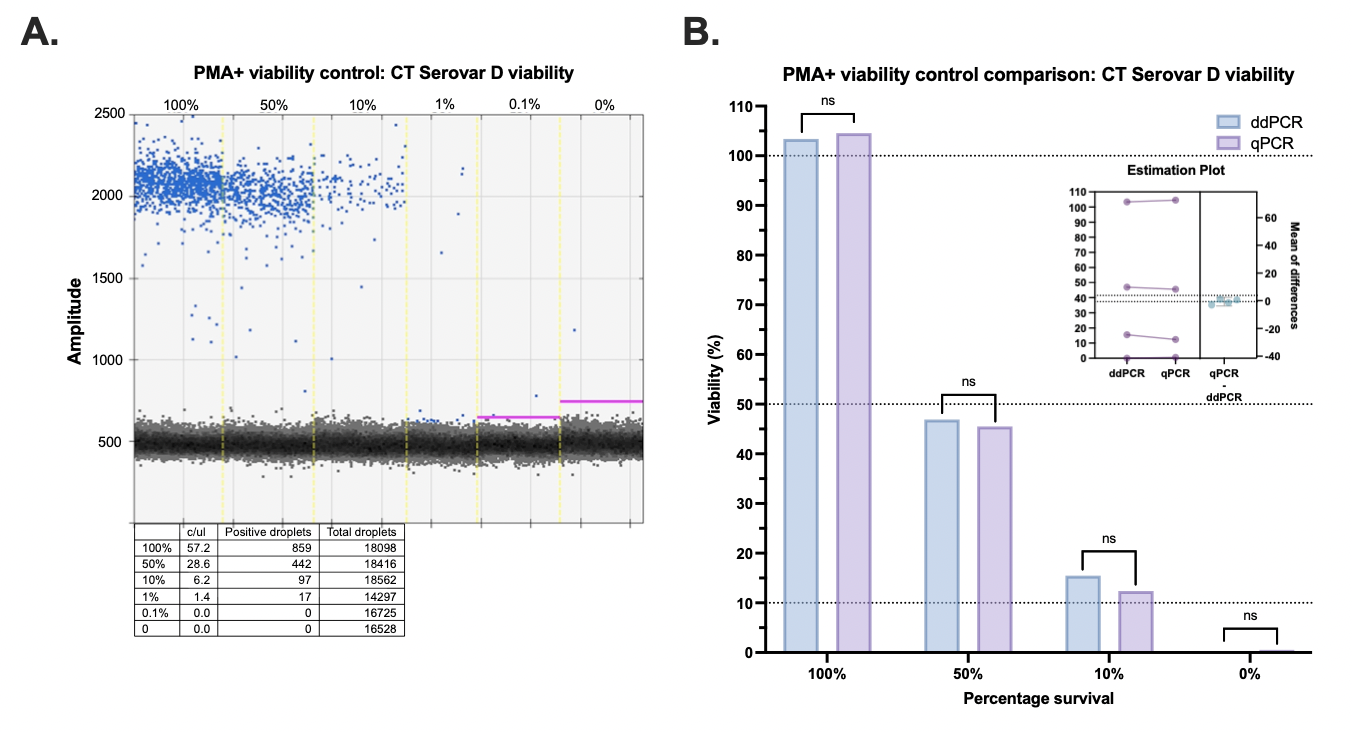


**Supplementary Figure 4. Assessing viability using droplet digital PCR. A.** Viability gradient of *Chlamydia trachomatis* (CT, Serovar D). The percentage (x-axis) represents proportion of living bacteria in a sample treated with photoreactive propidium monoazide (PMA). Concentration is derived from the number of positive droplets and given in copies per μL (c/ul). Note that only PMA+ samples are shown, and that viability is calculated based on the ratio between PMA+ and PMA- samples. Pink lines represent manual thresholds where automatic software detection failed to include positive droplets. **B.** Viability derived from ddPCR (blue) and qPCR (purple) for the control strain CT Serovar D. No statistically significant difference (two-tailed parametric t-test) between the methods can be observed (estimation plot, top right).

**Supplementary Table 1. Differences between clinic based routine PCR, qPCR and ddPCR**

|  | **Clinic CT EC** | **qPCR**  **CT EC** | **ddPCR [DNA] EC** | **V%** | **PCR + Gel EC** | **Clinic**  **CT RC** | **qPCR**  **CT RC** | **ddPCR [DNA] RC** | **V%** | **PCR + Gel RC** | **Clinic**  **CT PA** |
| --- | --- | --- | --- | --- | --- | --- | --- | --- | --- | --- | --- |
| **NL15** | 25.8 | NEG | NEG | NA | POS | 24.4 | NEG | 6.1E+02 | 3 | POS | NA |
| **NL6** | 28.4 | NEG | NEG | NA | POS | 26.3 | NEG | 3.6E+04 | 0 | POS | NA |
| **NL55** | 36.2 | NEG | NEG | NA | NEG | NEG | NA | NEG | NA | NEG | NEG |
| **NL81** | 14.6 | NEG | 3.9E+06 | 38 | POS | 28.4 | NEG | NEG | NA | POS | 32.0 |
| **NL152** | 20.1 | NA | 9.2E+04 | 19 | POS | 36.9 | NA | NEG | NA | NEG | 31.6 |
| **NL154** | NEG | NA | NEG | NA | NEG | 31.8 | NA | NEG | NA | NEG | 36.7 |
| **NL56** | 22.2 | 27,18 | 1.8E+04 | 61 | POS | NEG | NA | 3.4E+02 | 0 | POS | NEG |
| **LL131** | 14.6 | NA | 4.4E+06 | 40 | NA | NEG | NA | 1.2E+03 | 0 | NA | 38.3 |

**Supplementary Table 2. Primers for conventional PCR and MLST typing**

| **Name** | **Sequence** | **Function** |
| --- | --- | --- |
| hctB39 (forward) | 5 ́-CTCGAAGACAATCCAGTAGCAT-3 ́ | Outer primer *hctB* |
| hctB794 (reverse) | 5 ́-CACCAGAAGCAGCTACACGT-3 ́ | Outer primer *hctB* |
| CT046 (forward) | 5 ́-AACTCCAGCTTTTACTGCTA -3 ́ | Inner primer *hctB* |
| CT046 (reverse) | 5 ́-CCCCAAATATGCAACAGGAT-3 ́ | Inner primer *hctB* |
| CT222 (forward) | 5 ́-CTTTTCTGAGGCTGAGTATGATTT-3 ́ | Outer primer *CT058* |
| CT058 (reverse) | 5 ́-AATCYTCCTTRGCCTCTCTT-3 ́ | Outer primer *CT058* |
| CT058 (forward) | 5 ́-AGGTGGCTGCGTTAAGATAACT-3 ́ | Inner primer *CT058* |
| CT058 (reverse) | 5 ́-AAATTVGCCTGAAGTAGAGACA-3 ́ | Inner primer *CT058* |
| CT144:248 (forward) | 5 ́-ATGATTAACGTGATTTGGTTTCCTT -3 ́ | Outer primer *CT144* |
| CT144:1046 (reverse) | 5 ́-GCGCACCAAAACATAGGTACT-3 ́ | Outer primer *CT144* |
| CT144 (forward) | 5 ́-CGAAATCGGATATCTCTTTT-3 ́ | Inner primer *CT144* |
| CT144 (reverse) | 5 ́-CCTAAACATACGGCTATTCC-3 ́ | Inner primer *CT144* |
| CT172 (forward) | 5 ́-GATCAAGCCATCTTAGACATGC-3 ́ | Outer primer *CT172* |
| Four610 (reverse) | 5 ́-CGTCATTGCTTGCTCGGCTT-3 ́ | Outer primer *CT172* |
| CT172 (forward) | 5 ́-AGGTCGCCCAAATTCCATGT-3 ́ | Inner primer *CT172* |
| CT172 (reverse) | 5 ́-GCTCCGGCTATTTTGTTTAGGA-3 ́ | Inner primer *CT172* |
| pbpB1 (forward) | 5 ́-TATATGAAAAGAAAACGACGCACC-3 ́ | Outer primer *pbpB* |
| pbpB1 (reverse) | 5 ́- AAGAACCTTCCATCTCCTGAAT -3 ́ | Outer primer *pbpB* |
| CT682 (forward) | 5 ́-TCATCACTTTGCGTATATGGCA-3 ́ | Inner primer *pbpB* |
| CT682 (reverse) | 5 ́-AAAAGCTTRCKTACTTGATCGA-3 ́ | Inner primer *pbpB* |
| 118 (forward) | 5 ́-ATTGCTACAGGACATCTTGTC-3 ́ | Outer primer *ompA* |
| 1163 (reverse) | 5 ́-CGGAATTGTGCATTTACGTGAG-3 ́ | Outer primer *ompA* |
| ctr200 (forward) | 5 ́-TTAGGIGCTTCTTTCCAATAYGCTCAATC-3 ́ | Extra sequencing primer *ompA* |
| Ctr254 (reverse) | 5 ́-GCCAYTCATGGTARTCAATAGAGGCATC-3 ́ | Extra sequencing primer *ompA* |
| MOMP87 (forward) | 5 ́-TGAACCAAGCCTTATGATCGACGGA-3 ́ | Inner primer *ompA* |
| RVS1059 (reverse) | 5 ́-GCAATACCGCAAGATTTTCTAGATTTCATC-3 ́ | Inner primer *ompA* |
